# Virtual Physical Education During COVID-19: Exploring Future Directions for Equitable Online Learning Tools

**DOI:** 10.3389/fspor.2021.716566

**Published:** 2021-08-26

**Authors:** Emily M. D'Agostino, Mark Urtel, Collin A. Webster, Jaimie McMullen, Brian Culp

**Affiliations:** ^1^Department of Family Medicine and Community Health, Duke University School of Medicine, Durham, NC, United States; ^2^Department of Orthopaedic Surgery, Duke University School of Medicine, Durham, NC, United States; ^3^Department of Kinesiology, Indiana University Purdue University Indianapolis, Indianapolis, IN, United States; ^4^Department of Physical Education, University of South Carolina, Columbia, SC, United States; ^5^School of Sport and Exercise Science, University of Northern Colorado, Greeley, CO, United States; ^6^Department of Health Promotion and Physical Education, Kennesaw State University, Kennesaw, GA, United States

**Keywords:** virtual tools, online physical education, health equity, youth, school, students, COVID-19, diversity equity inclusion

## Abstract

**Introduction:** School closures prompted by the COVID-19 pandemic reduced opportunities for US youth to be physically active and disproportionately impacted health disparities in this population. Physical education provides the largest intervention to support the physical activity of school-aged youth, but teachers' opinions about how to maintain quality programming during virtual learning periods remain unexplored. Applying a diversity, equity and inclusion framework, this study explored physical education teachers' perceived significance of different design features for an online teaching tool to promote physical activity equity during school closures.

**Methods:** Previous literature and focus groups informed the development of a survey administered in summer/fall 2020. Survey participants (*n* = 60) were physical education teachers from 400 randomly selected US preschool-12th grade schools drawing from a national database. Participants rated the significance of four design features in relation to five key attributes of an online supplement to in-person physical education programs. One-way ANOVAs were used to assess differences in teachers' ratings by demographic characteristics.

**Results:** Between-group differences were found in teacher ratings of design features related to the usability, accessibility, equitability, and formal assessment capabilities of an online physical education tool. Differences were based on teacher gender, school level, and geographic location.

**Conclusions:** Future research to promote physical activity equity among preschool-12th grade youth should examine tailored virtual physical education learning tools that address what teachers perceive to be the most significant design features to support equitable physical education among diverse student groups.

## Introduction

The disproportionate impact of coronavirus disease 2019 (COVID-19) on vulnerable populations has highlighted grave health inequities (Berkowitz et al., 2020), particularly among marginalized youth (Fortuna et al., 2020; Morales et al., 2020). School settings provide youth with critical opportunities for physical activity (PA), a key driver of positive physical, social-emotional and mental health among youth (WHO, 2020). However, recent reports document PA-related effects of the COVID-19 pandemic on US school-aged youth, including decreases in PA participation and increases in sedentary behavior during home learning periods compared with prior to the COVID-19 pandemic (Dunton et al., 2020). COVID-19 also has indirect effects on minority and low-income youth, including exacerbated poverty, learning losses, poorer social-emotional and mental health, and higher school dropout rates attributable to school closures (Christakis et al., 2020; Dorn et al., 2020; Schulz et al., 2020; Benfer et al., 2021).

School physical education represents the largest youth PA intervention worldwide, given that physical education is a compulsory subject in many school curricula. Although usual in-person physical education programming is not without challenges (Hardman, 2008), school closures due to COVID-19 created a new host of obstacles. As preschool through 12th grade (P-12) physical education shifted to virtual learning platforms (Webster et al., 2021), physical education teachers and administrators were swiftly required to deliver robust virtual programs without adequate training and provision of appropriate teaching and learning resources. Online learning is, by its own nature, inequitable for school-aged youth, due in part to unequal access to technology, consistent high speed internet, adult supervision and support, sports equipment, and physical space to participate in online physical education (Daum, 2020). Additional inequities are presented for youth with disabilities who are particularly dependent on school physical education for PA engagement, and face barriers to being physically active in home environments (Esentürk, 2020).

If designed appropriately, online physical education may have the potential to reduce health disparities related to inequitable opportunities for PA engagement (Draper et al., 2021). The perspectives of physical education teachers are paramount to the design of online physical education resources that optimally supplement and support quality in-person programming, taking into consideration the diverse learning and PA needs of students. In light of pervasive inequities in youth PA, this study applied a diversity, equity and inclusion framework (Dashper and Fletcher, 2013). The aim of the current study was to examine US P-12 physical education teachers' perceived significance of different design features for an online teaching tool to promote PA equity during school closures. This study was developed as an exploratory pilot investigation to inform next steps toward the development of such a tool for professional practice and applied research in the area of equitable online physical education.

## Materials and Methods

### Participants and Sampling Procedure

Participants in this study included 60 current P-12 physical education teachers representing all regions of the US. Originally, 400 P-12 schools were identified using a proportionate random sampling technique as harvested from the Institute of Education National Center for Education Statistics (IES-NES) database (https://nces.ed.gov/ccd/schoolsearch/). This database includes all schools in the US listed by state. The list of schools for each state spans numerous webpages with ~10 schools included per page. Schools from each state and the District of Columbia were selected based on the percentage makeup of each state (i.e., number of webpages listing schools for the state) to the total number of pages of schools listed on the IES-NCES website. For example, there were 89 pages listing schools for Alabama. This represented 0.01% of the total number of pages for all schools listed on the website. Accordingly, three pages were randomly selected to represent Alabama schools, and one school from each of these three pages was randomly selected to search for physical education teachers' email addresses. Email addresses for all physical education teachers listed on a given school website were obtained. For any school that was identified as a charter or magnet school, or in cases where no email address for a physical education teacher could be found, another school from the same page was randomly selected. If physical education teacher emails could not be obtained from any of the schools listed on a page, another page was randomly selected. A blanket invitation to participate in the study was sent to all procured email addresses asking recipients to complete an electronic survey. There were no incentives offered to complete the survey. From the initial email, there were 18 “failure to deliver” automated replies; additionally, three prospective participants replied that they were unable to complete the survey (district/corporation policy). A reminder email was sent one week later. In all, individuals invited to participate had 14 days to complete the survey. The final response rate for the survey was 16%. This study was approved by the Indiana University Institutional Review Board.

### Survey Design

The research team developed an electronic survey for this study (Appendix A) using Qualtrics, based on previous literature (Smith and Boling, 2009) and the results of three focus group interviews the team conducted with P-12 physical education teachers. Convenience sampling was used to recruit nine teachers for the focus groups. Each interview lasted ~75 min. Interviews focused on the teachers' experiences and recommendations with respect to online physical education, particularly in reference to the recent shift in virtual teaching and learning. The interviews were held virtually and recorded via Zoom, and then transcribed verbatim. Transcripts were qualitatively analyzed using inductive analytic methods to identify patterns in the data. Overall, five key attributes of online physical education were used to frame the survey: usability, accessibility, equitability, facilitation of formal assessment, and capacity for PA tracking. Specifically, teachers described how the pivot to remote learning could marginalize a particular set of students. Examples provided from the teachers included students with disabilities, students living in multi-generational households at a low socio-economic status, students from households with English as a new language, and racial/ethnic minority student populations. Additionally, four design features specific to organizing and providing online resources were identified as potentially helpful in addressing each attribute: a bank of videos for teachers to learn from, a bank of videos for students to view, a listing of activities cataloged by learning standards, and a discussion board for teachers. The survey included an item for each key attribute (including a definition of the attribute and relevant examples), asking participants to rate the significance of each of the 4 aforementioned design features to enhancing the attribute. A rating scale of 0–100 was used with 0 indicating “not at all significant” and 100 indicating “very significant.” Additional items were included on the survey to gather information about participant demographics, including gender, age range, school level (elementary, middle school, or high school), geographic location (coastal or non-coastal), and years of teaching experience.

### Statistical Analysis

Descriptive statistics were calculated for participant demographics and significance ratings of each design feature by key attribute. Then, for each key attribute, separate one-way analyses of variance (ANOVA) were performed to assess differences in significance ratings for each design feature by teacher demographics (gender, age, school level, geographic location, years of teaching experience). SPSS software (version 27) was used, and a 0.05 significance level was applied for all models.

## Results

Participant demographics are presented in Table 1. Participants were 52% male, mostly between 35–54 years (61%), 58% non-coastal geographic locations, and mostly elementary school level teachers (62%; mean [SD] years of teaching experience: 17.4 [11.1]).

**Table 1:** Demographics of survey respondents (*n* = 60).

	***n***	**%**
Gender		
Female	29	48
Male	31	52
Age range (years)		
25–34	10	17
35–44	20	33
45–54	17	28
55+	13	22
Geographic location		
Coastal	25	42
Non-coastal	35	58
School level		
Elementary	37	62
Middle grades	13	22
High School	10	16
Teaching experience (years)	17.4	*SD* = 11.1

*SD, standard deviation*.

The median significance ratings for each design feature by key attribute are presented in Table 2. Across participants, a bank of videos for students to view was rated the highest in relation to the usability (86.0), accessibility (86.5), and assessment feasibility (81.5) of an online physical education tool. Regarding the equitability of such a tool, participants rated a discussion board for teachers as the most significant design feature (82.0). A list of activities cataloged by standards was rated as the most significant design feature with respect to being able to track students' PA (90.0).

**Table 2:** Median with interquartile range scores of each design feature by key attribute.

		**Gender**		**Age group (years)**				**Geographic location**		**School level**		
**Median (IQR)**	**Overall**	**F**	**M**	**25–34**	**35–44**	**45–54**	**55+**	**Non-coastal**	**Coastal**	**EL**	**MG**	**HS**
**Usability**												
Bank of videos for teachers to learn from	81 (26.5)	82 (28)	80 (30)	90 (32)	80.5 (20)	75 (37)	81 (30)	81 (37)	81 (24)	85 (27)	78 (29)	80 (41)
Bank of videos for students to view	86 (28)	87 (24)	85 (30)	90 (28.5)	89 (22.5)	85 (24.5)	76 (38.0)	90 (28)	80 (23)	80 (26.5)	91 (33.5)	90 (39)
List of activities cataloged by standards	82.5 (29.5)	83 (27)	80 (35)	85 (31.2)	84 (26)	80 (21.5)	79 (39)	87 (29)	80 (30)	80 (27.5)	88.5 (23.7)	75 (60)
Discussion board for teachers	85 (46.2)	90 (48.5)	85 (40)	87.5 (51)	95 (38.5)	85 (35.5)	80 (56.5)	90 (50)	85 (37)	79 (50)	90.5 (35)	85 (42)
**Accessibility**												
Bank of videos for teachers to learn from	80 (38.5)	80 (39)	80 (39)	83 (39.2)	84.5 (47.2)	84 (40)	75 (36)	84 (39)	77 (45.5)	77 (46)	82 (17.2)	79 (40)
Bank of videos for students to view	86.5 (40)	80 (44)	90 (40)	90 (39.2)	97.5 (36.2)	80 (35)	72 (30.5)	90 (25)	72 (49.5)	80 (40)	87.25 (23.2)	90 (50)
List of activities cataloged by standards	82 (39.7)	80 (31)	90 (40)	85 (41.2)	84 (28.7)	81 (41)	82 (38.5)	89 (39)	82 (43.5)	80 (42.5)	90 (19.7)	95 (40)
Discussion board for teachers	77.5 (45)	76 (45)	79 (33)	67.5 (50)	90 (41.5)	75 (50.5)	79 (41)	71 (41)	90 (46)	79 (44)	85 (26.7)	67 (52.5)
**Equitability**												
Bank of videos for teachers to learn from	80 (44)	75 (50)	81 (37)	69 (55)	80.5 (30)	81 (47.5)	65 (35.5)	85 (42)	69 (32.5)	75 (44.5)	85 (26)	75 (55)
Bank of videos for students to view	76 (30.7)	77 (30.5)	75 (41)	72 (42.5)	76 (29)	77 (47.5)	70 (23.5)	87 (36)	70 (25.5)	75 (30)	90 (18.7)	67 (38)
List of activities cataloged by standards	78 (44.5)	80 (41)	70 (50)	68 (62.7)	82.5 (54.5)	80 (46.5)	76 (33.5)	88 (49)	70 (37.5)	80 (39.5)	94.5 (23.5)	51 (48.5)
Discussion board for teachers	82 (41.2)	75 (44)	85 (40)	86 (54.5)	84 (30.7)	76 (42)	85 (42)	76 (49)	85 (40)	76 (45.5)	92 (28.5)	85 (47)
**Assessment**												
Bank of videos for teachers to learn from	81 (33.7)	83 (17.5)	70 (50)	70 (44)	77.5 (25.2)	90 (35.5)	77 (39.5)	82 (37)	80 (31)	77 (30.5)	85.5 (21)	75 (55)
Bank of videos for students to view	81.5 (35.7)	84 (51)	70 (50)	84.5 (40.7)	80 (33.2)	90 (32)	75 (52)	79 (35)	84 (33)	80 (38)	89.5 (17.2)	80 (55)
List of activities cataloged by standards	79 (40.5)	80 (30)	75 (47)	95 (52)	70 (38)	78 (48.5)	89 (30)	75 (49)	84 (30)	78 (47.5)	89.5 (41)	70 (80)
Discussion board for teachers	80.5 (43.2)	80 (39)	83 (40)	95 (45.7)	75 (36)	81 (54)	80 (32)	70 (49)	90 (27.5)	80 (49)	88.50 (25.2)	75 (64)
**Tracking PA**												
Bank of videos for teachers to learn from	75.5 (50)	76 (45)	70 (52)	87.5 (48.5)	71 (64)	81 (50)	69 (39)	81 (50)	71 (53.5)	70 (51)	91 (29.2)	76 (29.2)
Bank of videos for students to view	77 (48)	78 (44.5)	75 (50)	77.5 (44)	77 (63)	80 (49.5)	70 (38)	76 (50)	80 (36)	75 (49)	93.5 (21.2)	69 (45.5)
List of activities cataloged by standards	90 (29.7)	90 (25)	90 (45)	100 (13.7)	86.5 (27.2)	90 (47.5)	76 (38)	88 (29)	90 (37.5)	81 (30)	95.5 (16.2)	90 (45)
Discussion board for teachers	77 (49.2)	75 (48.5)	79 (50)	90 (38.5)	74.5 (46.5)	70 (54)	80 (33.5)	71 (50)	80 (44)	75 (48.5)	86.5 (35.7)	74 (68.5)

*ES, elementary school; F, female; HS, high school; IQR, interquartile range; M, male; MS, middle school; PA, physical activity*.

When considering demographic variables, there was a statistically significant interaction effect for the usability attribute, F_(2, 57)_ = 4.57, *p* = 0.04. Main effects analysis revealed a significant effect for gender (*p* < 0.01) with higher ratings among female teachers than male teachers for the design feature of listing standards-aligned activities. There was also a statistically significant interaction effect for the accessibility attribute, *F*_(3, 56)_ = 10.50, *p* < 0.01, with follow-up analysis again indicating a significant effect for gender (*p* = 0.03). Specifically, male teachers rated a simple listing of activities lower than female teachers in relation to the usability attribute.

Additionally, there was a significant interaction effect for the equitability attribute, *F*_(3, 57)_ = 5.00, *p* = 0.03. Main effects analysis showed a significant effect for region (*p* < 0.01) with teachers in non-coastal locations rating the bank of videos for students to view that could be directly used in their teaching higher than teachers in coastal locations. Finally, there was a significant interaction effect for the key attribute of facilitating formal assessment, F_(3, 57)_ = 5.53, *p* = 0.03, with follow-up analysis demonstrating a significant effect for school level (*p* = 0.01). High school teachers rated the bank of videos for students to view that could be directly used in their teaching at a lower level of significance than middle school or elementary school teachers.

## Discussion

In order to promote accessible and equitable online physical education learning to increase PA engagement, intentional, systematic approaches must be adopted that support the needs of P-12 physical education teachers. Based on a stratified national random sample, this pilot work drew upon the perspectives of physical education teachers to better understand which design features may best support the efficacy of an online physical education tool's key attributes.

Teachers' prioritization of different design features for certain attributes varied across gender (usability and accessibility), geographic location (equitability), and school level (facilitation of formal assessment). Promoting access to PA in diverse home environments will require tailoring and tool flexibility to meet the needs of P-12 physical education teachers and their students. These findings have implications for the development of equitable online physical education instructional resources to optimally support P-12 physical educators across different teacher populations and regions. Specifically, physical educators seek robust and nimble online resources that have the capability to be tailored to their particular contextual and student needs, so they can continue to serve all students even when in-person programming is not possible. In order to address systematic inequities that prevent participation in remote physical education learning, customizable tools that support educators in developing their own content or modifying existing content may be necessary. Future research in this area should examine ways to promote access and participation in online physical education amongst diverse student groups through supporting educators with supplemental online materials based on youth access to parent/sibling supervision, safe/spacious indoor/outdoor settings, equipment, self-efficacy and ability.

Although online resources are already available for educators, they are not sufficient to meet current physical education teacher needs. For example, recent literature determined that “student access to online learning” and “availability of teacher resources” were substantial challenges related to online physical education instruction during the COVID-19 pandemic (Pavlovic et al., 2021). Similarly, Mercier et al. reported that 20% of physical education teachers felt less effective teaching their students online during the pandemic. The authors inferred that teacher responses may not reflect actual learning given that half of the sample did not use assignments or video instruction (Mercier et al., 2021). Moreover, a scoping review by Killian et al. found that no prior research has evaluated the efficacy of online and blended instruction to promote or inhibit skill development or promote PA outside of school (Killian et al., 2019). There is a clear need for further research to inform the development of customizable online learning tools that address physical education teacher needs, particular during periods of virtual instruction.

This study is strengthened by data drawn from a randomly selected, national sample of P-12 physical education teachers representing 400 schools, and with heterogeneity across teacher age, gender, years of experience, teaching level, and geographic region. Limitations include non-response bias from invited participants who opted not to enroll in the study, and inability to account for key sociodemographic factors that are related to developing equitable online P-12 physical education tools, including teacher race/ethnicity, urban/rural setting, and demographics of students taught (Evans, 2014; Hodge et al., 2017). Additionally, as this study was exploratory, further research will benefit from further development and validation of methods that best capture the diverse needs of teachers and their students within the context of online physical education.

## Conclusion

The COVID-19 pandemic has had disproportionate impacts on US school-aged youth health disparities related to reduced opportunities for equitable PA. This preliminary study found higher prioritization of different design features for certain attributes across gender, geographic location, and school level for supporting standards-based, accessible, and equitable PA based on a national sample of P-12 physical education teachers. Pilot testing of an online tool that provides customizable physical education learning activities is a suitable next step for research to examine potential to promote PA engagement for diverse student groups. This tool has implications for supporting teachers in promoting PA equity among youth during COVID-19 and other times of virtual learning by offering learning models that can cater to different student populations with varied access to technology, resources, supervision, and equipment. Promoting equitable access to routine daily PA has potential to promote overall health equity related to preventing chronic conditions (cardiovascular disease, asthma, diabetes, obesity, and metabolic syndrome) (Biddle et al., 2004; Physical Activity Guidelines Advisory Committee, 2008; Gordon-Larsen et al., 2010; Tammelin et al., 2014; Yang et al., 2014), as well as supporting mental health and reducing stress associated with traumas related to social isolation during the COVID-19 pandemic (Loades et al., 2021).

## Data Availability Statement

The raw data supporting the conclusions of this article will be made available by the authors, without undue reservation.

## Ethics Statement

The studies involving human participants were reviewed and approved by Indiana University Institutional Review Board. Written informed consent for participation was not required for this study in accordance with the national legislation and the institutional requirements.

## Author Contributions

For this manuscript, all authors co-designed and implemented the study. MU conducted the analysis. EMD, MU and CAW prepared all sections of the text. All authors reviewed all sections of the text and approved the final version of this manuscript.

## Conflict of Interest

The authors declare that the research was conducted in the absence of any commercial or financial relationships that could be construed as a potential conflict of interest.

## Publisher's Note

All claims expressed in this article are solely those of the authors and do not necessarily represent those of their affiliated organizations, or those of the publisher, the editors and the reviewers. Any product that may be evaluated in this article, or claim that may be made by its manufacturer, is not guaranteed or endorsed by the publisher.
