# Supplementary material for: Virtual Physical Education During COVID-19: Exploring Future Directions for Equitable Online Learning Tools
Source: Front Sports Act Living. 2021 Aug 26;3:716566. doi: 10.3389/fspor.2021.716566 (PMC8426569; doi:10.3389/fspor.2021.716566)
Supplement: Supplementary file 1 [file Data_Sheet_1.pdf]

Appendix A

Survey items (without the slider scale that was grided by 10 from 0-100).

1. Which of the below design features, in your opinion, would make an online supplement for physical education user friendly and easy to navigate. (Slide for significance: 0 = Not at all significant and 100 = Very significant)

*Bank of videos that you, as the teacher, could select to learn from (i.e., a 3-5 minute video from an expert on a specific topic on online teaching).

*Bank of videos that you, as the teacher, could select for your students to view (i.e., a 3-5 minute video for a student to follow along with as an element of a larger lesson).

*Listing of activities you could offer your students, cataloged by a national or state standard for physical education.

*An up-to-date discussion board for teachers to offer updates on what is working and what is not; categorized by various topics (instant activities, physical activity tracking, etc.)

2. Which of the below design features, would be helpful in terms of making it appropriate to serve students in *various living environments* [access to open spaces, access only to a single indoor room, limited to no equipment, etc.]. (Slide for significance: 0 = Not at all significant and 100 = Very significant)

*Bank of videos, from experts, discussing how to adapt a lesson to various home environments and spaces for moving (i.e., single room, backyard, limited equipment).

*Bank of videos modeling physical education in various home environments and spaces for moving.

*Listing of activities catalogued or listed per various home environments.

*An up-to-date discussion board for teachers to offer updates on what is working and what is not; categorized by various home environments (i.e., single room, backyard, limited equipment).

3. Please list or describe what design features would be helpful to serve *the needs of diverse learners* (e.g., different ages, skill levels, abilities, cultural backgrounds).

*Bank of videos, from experts, discussing how to differentiate a lesson for diverse learners (i.e., age levels, students with varying ability levels, or English as a new language learners).

*Bank of videos modeling physical education being delivered, meaningfully and respectfully, to diverse learners.

*Listing of activities addressing various characteristics of diverse learners.

*An up-to-date discussion board for teachers to offer updates on what is working and what is not as it relates to strategies to meaningfully and respectfully engage diverse learners.

4. Which of the following design features would be helpful to you so you could implement *formal assessments* to gauge student learning and guide further instruction.

*Bank of videos, from experts, discussing how you could possibly assess remote teaching and learning.

*Bank of videos modeling how you could assess teaching and learning remotely.

*Listing of activities addressing various methods of assessing learners remotely.

*An up-to-date discussion board for teachers to offer updates on what is working and what is not as it relates to strategies to meaningfully and respectfully engage diverse learners.

5. Please list or describe what design features would be helpful for you in order to meaningfully track students' physical activity participation levels in your physical education class/program.

*Bank of videos, from experts, discussing how you could possibly track physical activity levels of students in this remote environment.

*Bank of videos modeling how you could track physical activity levels of your students, remotely.

*Listing of ways you can track physical activity levels of your students remotely.

*An up-to-date discussion board for teachers to offer updates on what is working and what is not as it relates to tracking physical activity levels of their students, remotely.
